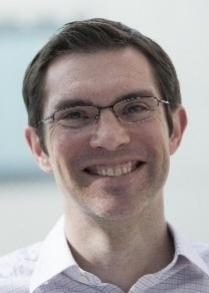# Rediscovering Sulfinylamines as Reagents for Organic Synthesis

**DOI:** 10.1002/chem.202100321

**Published:** 2021-06-01

**Authors:** Thomas Q. Davies, Michael C. Willis

**Affiliations:** ^1^ Chemistry Research Laboratory University of Oxford 12 Mansfield Road Oxford OX1 3TA UK

**Keywords:** cycloadditions, sulfinylamines, sulfoximines, sulfonimidamides, sulfur

## Abstract

Sulfinylamines (R−N=S=O), monoaza analogues of sulfur dioxide, have been known for well over a century, and their reactivity as sulfur electrophiles and in Diels‐Alder reactions is well‐established. However, they have only rarely been used in organic synthesis in recent decades despite the increasing prominence of compounds containing N=S=O functionality, such as sulfoximines and sulfonimidamides. This Minireview aims to bring wider visibility to the unique chemistry enabled by this class of compounds. We focus on advances from the last 10 years, including the first examples of their use in the one‐pot syntheses of sulfoximines and sulfonimidamides. Also covered are the reactions of sulfinylamines with carbon‐centred radicals, their use for formation of heterocycles through cycloadditions, and catalytic enantioselective allylic oxidation of alkenes *via* a hetero‐ene reaction. These examples highlight the different reactivity modes of sulfinylamines and their underappreciated potential for forming molecules which contain high‐ or low‐valent sulfur, or even no sulfur at all.

## Graphical abstract

**Recent applications of*****N*****‐sulfinylamines**: In this Minireview, the use of *N*‐sulfinylamines (R−N=S=O) in sulfoximine and sulfonimidamide synthesis, heterocycle formation and catalytic enantioselective oxidation of alkenes is covered. Brief discussions of their preparation, stability, properties and potential future applications are also included.

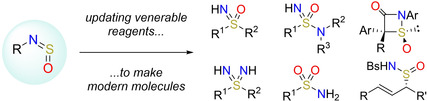

## Introduction

The surprisingly long history of sulfinylamines began in 1878 when Böttinger discovered that “thionyl chloride acts extremely violently on aniline … “every drop of the thionyl chloride solution produces a hissing noise”.^[1]^ Although the structure of the product was not reported in this publication, his vivid account fortunately did not deter future practitioners. In 1890 Michaelis and Herz revisited the reaction and confirmed the product as *N*‐sulfinylaniline, Ph−N=S=O **1**.^[2]^

**Scheme 1:**
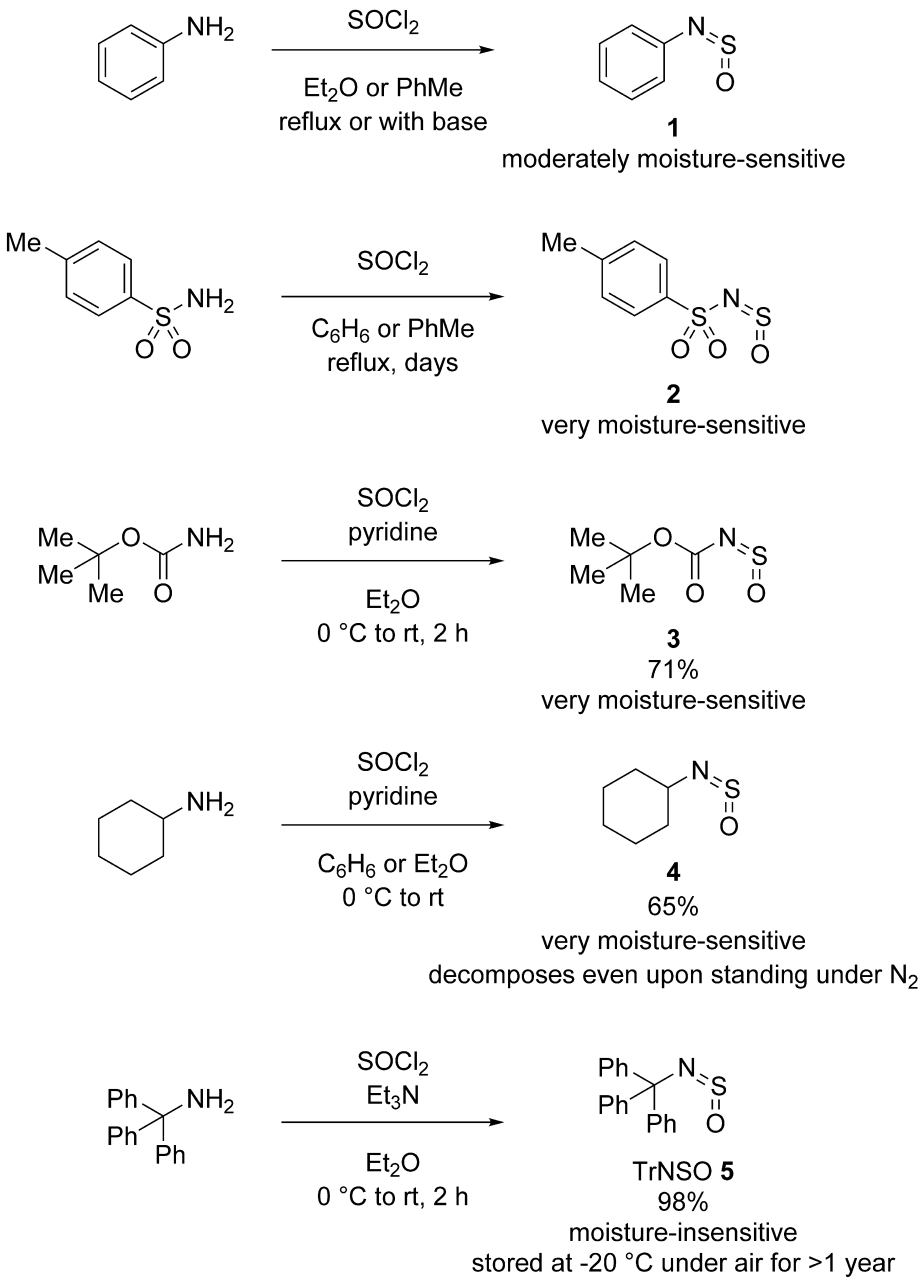
Preparation and properties of different classes of sulfinylamines.

Interestingly, the synthesis of sulfinylamines has not changed significantly in the intervening period. They are still most commonly prepared by the reaction of primary amines with thionyl chloride (Scheme 1). In the past this was often performed under refluxing conditions, and in the case of weakly nucleophilic sulfonamides high temperatures and long reaction times are necessary to achieve full conversion.^[3]^ For more reactive or sensitive amine derivatives, the reaction can be accomplished under milder conditions (0 °C or room temperature) in the presence of a base such as triethylamine or pyridine, the conjugate acid of which is then removed by filtration.^[4]^ For *N*‐sulfinylsulfonamides and carbamates, vacuum distillation is the typical method of purification. *N*‐Sulfinylanilines may also be purified by distillation, while the recently reported reagent *N*‐sulfinyltritylamine (TrNSO **5**) is typically obtained pure after filtration through Celite. Among other methods, the use of chlorosulfinylimidazole has been reported to have a notable advantage in the synthesis of *N*‐sulfinylsulfonamides, giving a 97 % yield of product in just 1 hour at room temperature.^[5]^ Less commonly used methods include the reaction of silylamines with SOCl_2_ ^[6]^ or SO_2_,^[7]^ or the transsulfinylation of *N*‐sulfinylsulfonamides with more nucleophilic amino compounds.^[3]^

The electrophilicity of sulfinylamines’ central tetravalent sulfur atom dominates their reactivity but may also be said to be their Achilles’ heel; many sulfinylamines are extremely moisture‐sensitive.^[3]^ This can make their purification challenging and is likely part of the reason why they have not been more widely used. However, the identity of the substituent on nitrogen strongly influences their reactivity and stability, and the judicious design of this group can result in stable reagents.

In the authors’ own experience, sulfinylamines bearing strong electron‐withdrawing groups such as tosyl (**2**) and Boc (**3**) quickly hydrolyse upon exposure to moist air, and as such must be handled under an inert atmosphere. Sulfinylamines derived from anilines (**1**) show markedly improved hydrolytic stability and may be manipulated under air for short periods, such as to remove solvent on a rotary evaporator, but must be stored under inert gas. *N*‐Sulfinylalkylamines with short alkyl chains were reported by Kresze to be “colourless liquids … [which] fume in moist air and gradually decompose even when air and moisture are excluded”. This accords with our own experience of preparing *N*‐sulfinylcyclohexylamine **4** using Kresze's procedure.^[3]^ The pure compound decomposed (turned black) when stored at room temperature overnight under nitrogen. We speculate that this may be due to formation of an oligomeric or polymeric species by intermolecular attack of the nucleophilic nitrogen atom at sulfur; indeed, the parent sulfinylamine, HNSO (thionylimine), is known to polymerise above −70 °C.^[8]^ Sterically demanding groups drastically increase stability, with *N*‐sulfinyltritylamine (TrNSO, **5**)^[9]^ able to be stored for at least a year under air with no appreciable decomposition.

Sulfinylamines exist seemingly exclusively in the (*Z*) form,^[10]^ with the oxygen on the same side as the nitrogen substituent, as drawn throughout this review. This preference outweighs steric repulsion, as seen in the X‐ray crystal structure of TrNSO.^[11]^ Our group carried out computational (Natural Bond Orbital) studies on the structure of the sulfinylamine Ph−O−N=S=O and found that favourable donation of the nitrogen lone pair into the S−O σ* orbital, and of the sulfur lone pair into the N−O σ* orbital, help to stabilise the (*Z*)‐conformation relative to the (*E*)‐conformation.^[12]^

**Scheme 2:**
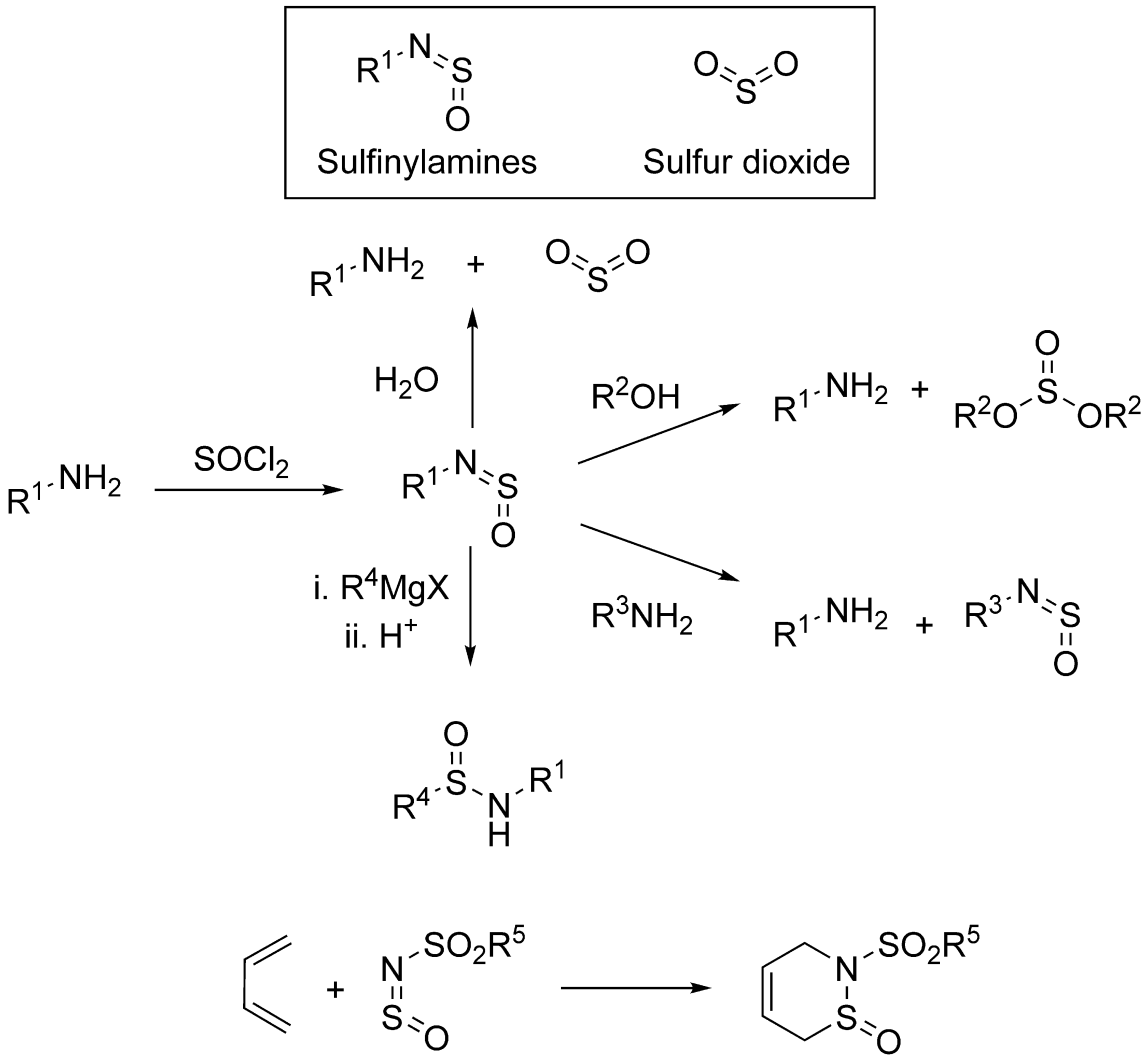
Some fundamental reactions of sulfinylamines.

The fundamental chemistry of sulfinylamines including Diels‐Alder cycloadditions^[13]^ and attack of various nucleophiles such as alcohols, amines and Grignard reagents^[14]^ (Scheme 2) has been covered in previous reviews and so will not be described in detail here.^[3]^ This Minireview will summarise advances principally from the last decade. The first part will describe the use of sulfinylamines as reagents for the preparation of medicinally relevant organosulfur compounds including sulfonimidamides and sulfoximines. The second section will focus on their recent use in cycloaddition and ene reactions, often providing valuable products with high selectivities.

## Synthesis of Sulfur(VI) Compounds

Our interest in sulfinylamines was sparked by considering their status as mono‐aza analogues of sulfur dioxide. SO_2_ can be used, often in the form of a solid surrogate such as DABSO (1,4‐diazabicyclo[2.2.2]octane‐*bis*(sulfur dioxide) adduct),^[15]^ to prepare sulfones,^[16]^ sulfonamides^[17]^ and other sulfonyl‐containing molecules.^[18]^ These compounds are ubiquitous in marketed drugs and agrochemicals. Their nitrogen‐containing analogues, sulfoximines[^19^, ^20^] and sulfonimidamides,^[21]^ are less well‐known but are increasingly finding use as bioactive molecules due to their potential for asymmetry at sulfur, basic nitrogen atom and favourable balance of physicochemical properties.[^22^, ^23^, ^24^, ^25^] We envisaged that sulfinylamines could provide a rapid and convenient route to these compounds by acting as “HN=S=O” equivalents. However, at the outset of the research programme (2015) there were no such broadly useful, stable sulfinylamines known. This section details the reagents we have developed since and their successful application to the synthesis of complex organosulfur compounds.

**Scheme 3:**
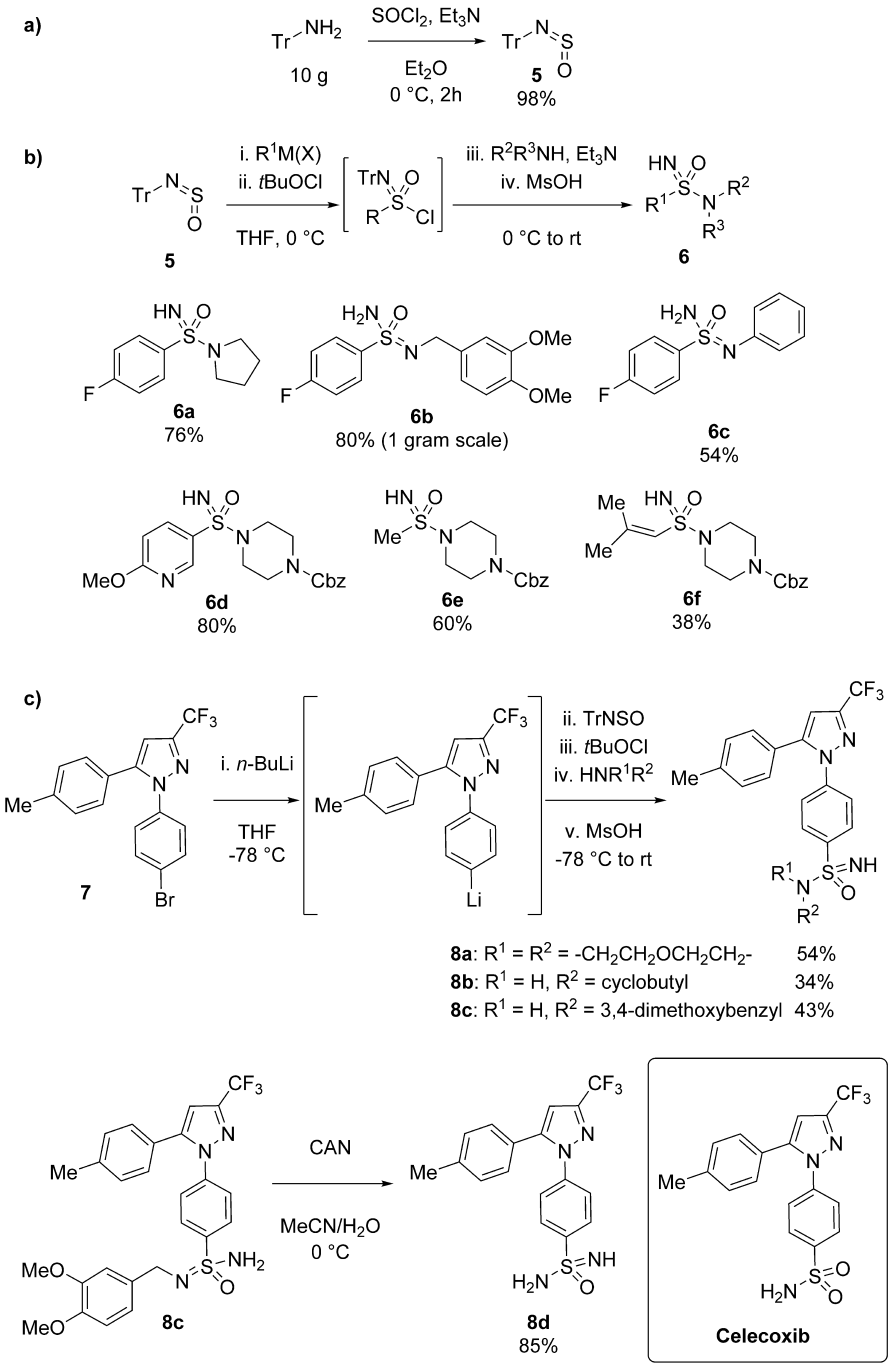
a) Decagram‐scale preparation of TrNSO **5**. b) One‐pot synthesis of NH‐sulfonimidamides from **5**. c) Synthesis of Celecoxib analogues.

Our first contribution to this area was the one‐pot, three‐component synthesis of sulfonimidamides from the combination of the sulfinylamine TrNSO^[11]^ **5**, Grignard reagents and amines (Scheme 3).^[9]^ As discussed earlier, TrNSO is a stable, solid reagent and is easily synthesised from tritylamine on 10 gram scale. Its stability is likely due to the steric bulk of the trityl group. Despite this, TrNSO reacts quantitatively with Grignard or organolithium reagents to give sulfinamides. These could then be oxidized *in situ*, using *tert*‐butyl hypochlorite (*t*‐BuOCl),^[26]^ to sulfonimidoyl chlorides, which were reacted with amines overnight at room temperature. The trityl group was removed using methanesulfonic acid (MsOH) to give NH‐sulfonimidamides in good yields (generally 60–80 %) over the one‐pot, four step process. This procedure represented the most direct route then known to prepare sulfonimidamides and worked with primary, secondary and aromatic amines, as well as alkyl, aryl and alkenyl nucleophiles. Analogues of the primary sulfonamide‐containing painkiller Celecoxib could be easily synthesised, demonstrating its application to medicinally relevant scaffolds (Scheme 3c). TrNSO has since been commercialised by several vendors, including Tokyo Chemical Industry, Sigma‐Aldrich, Key Organics and Cortex Organics.

**Scheme 4:**
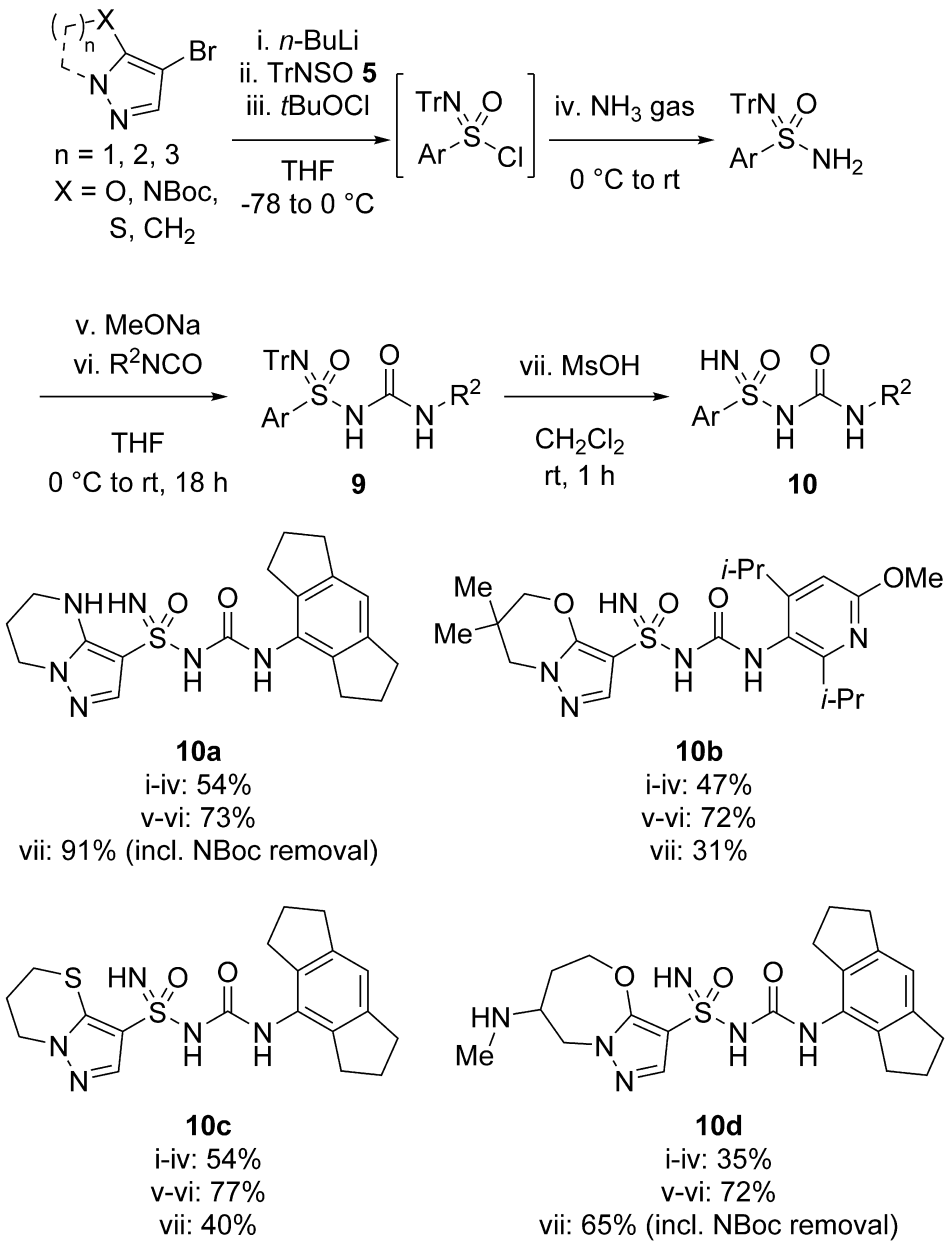
Synthesis of heteroarylsulfonimidoylureas from TrNSO **5**.

This method has recently been used by chemists at Genentech to prepare sulfonimidoylurea compounds as NLRP3 inhibitors.^[27]^ Heteroaryllithium reagents, generally made from 4‐bromopyrazole precursors, were prepared and reacted with TrNSO before chlorination with *t*‐BuOCl and addition of ammonia (Scheme 4). The trityl‐protected sulfonimidamides were then isolated and shown to react well with isocyanates to give complex sulfonimidoylureas **9**. Deprotection of the trityl group was effected with MsOH to give the final products **10**.

**Scheme 5:**
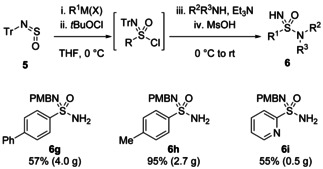
Preparative scale synthesis of sulfonimidamides from TrNSO, including incorporation of a challenging 2‐pyridyl unit.

TrNSO has also been used to prepare mono‐protected sulfonimidamides (**6 g**–**6 i**, Scheme 5) as substrates *en route* to an organocatalytic synthesis of enantiomerically enriched and deprotectable *N*,*N*,*N*’‐trisubstituted sulfonimidamides.^[28]^ Notably, over 4 grams of compound **6 g** was obtained in one reaction, and an excellent 95 % yield of compound **6 h** was achieved, giving 2.7 grams of product. A synthetically challenging and medicinally relevant unsubstituted 2‐pyridyl nucleophile was also incorporated in good yield (**6 i**).

**Scheme 6:**
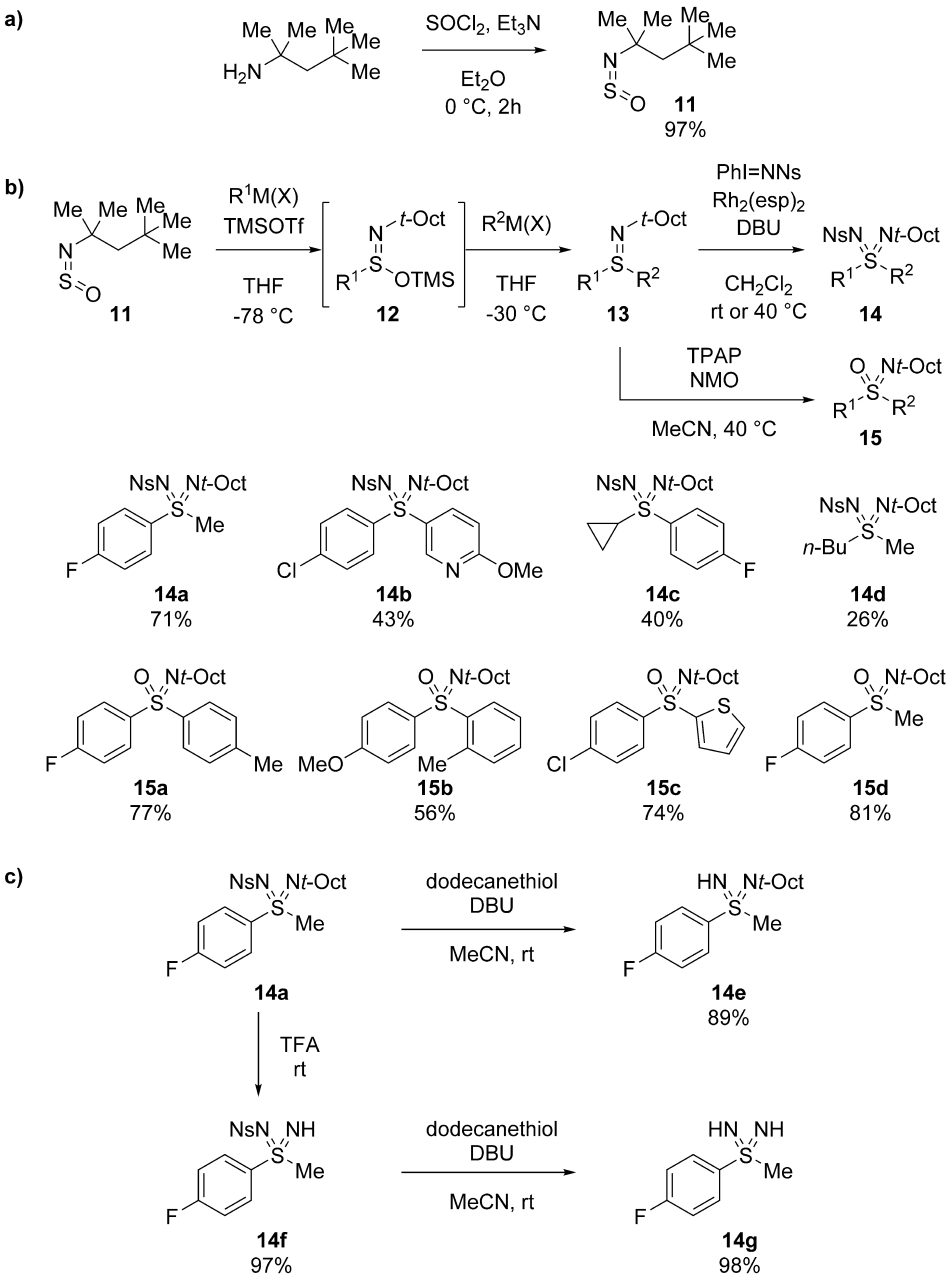
a) Preparation of *t*‐OctNSO **11**. b) Synthesis of sulfondiimines or sulfoximines by deoxygenation and subsequent Rh‐catalysed imination or Ley‐type oxidation. c) Selective removal of *t*‐octyl and nosyl protecting groups.

We considered that sulfinylamines could be used to prepare sulfondiimines, the diaza analogues of sulfones, through a sulfilimine intermediate by a deoxygenative transformation.^[29]^ This was achieved by the consecutive addition of trimethylsilyl triflate (TMSOTf) and a carbon‐centred organometallic reagent to *N*‐sulfinyl‐*tert*‐octylamine (*t*‐OctNSO **11**) at −78 °C to give *O*‐silylated intermediate **12** (Scheme 6). Subsequent addition of a second organometallic reagent at −30 °C gave *t*‐octyl‐protected sulfilimines **13** by substitution of the −OTMS group. The first example of metal‐catalysed imination on an N‐substituted sulfilimine was then developed using rhodium catalysis to give orthogonally N‐protected sulfondiimines **14**. Notably, the use of the pre‐formed iminoiodinane NsNIPh, instead of *in situ* formation by combining NsNH_2_ with PhI(OAc)_2_, was crucial to achieving high conversion as the basic sulfilimine intermediates can be protonated by the acetic acid by‐product of PhI(OAc)_2_. Under the optimal conditions, 1.3 equiv. of NsNIPh were combined with 2.5 mol% [Rh_2_(esp)_2_]_2_ in CH_2_Cl_2_ for 24 hours, giving sterically congested sulfondiimines in moderate to good yields. The highest yields were obtained for compounds containing one aryl and one alkyl substituent (**14 a**). Aryl‐aryl sulfilimines (**14 b**) were less reactive in the imination step, presumably due to their larger steric demand and loss of electron‐density at sulfur. On the other hand, dialkyl‐substituted sulfilimines proved to be relatively unstable, partially decomposing during work‐up and imination. Nevertheless, synthetically useful yields (20–35 %) of the sulfondiimines were still obtained (**14 d**). Sulfoximines **15** could also be prepared under Ley oxidation conditions (catalytic tetrapropylammonium perruthenate, TPAP, and *N*‐methylmorpholine‐*N*‐oxide, NMO), selectively oxidising the sulfur over nitrogen. Crucially, the nosyl and *t*‐octyl protecting groups could be selectively removed from the products. Sulfondiimines bearing *N*‐aryl, ‐allyl, ‐acetyl, and ‐benzyl groups were all accessible.

**Scheme 7:**
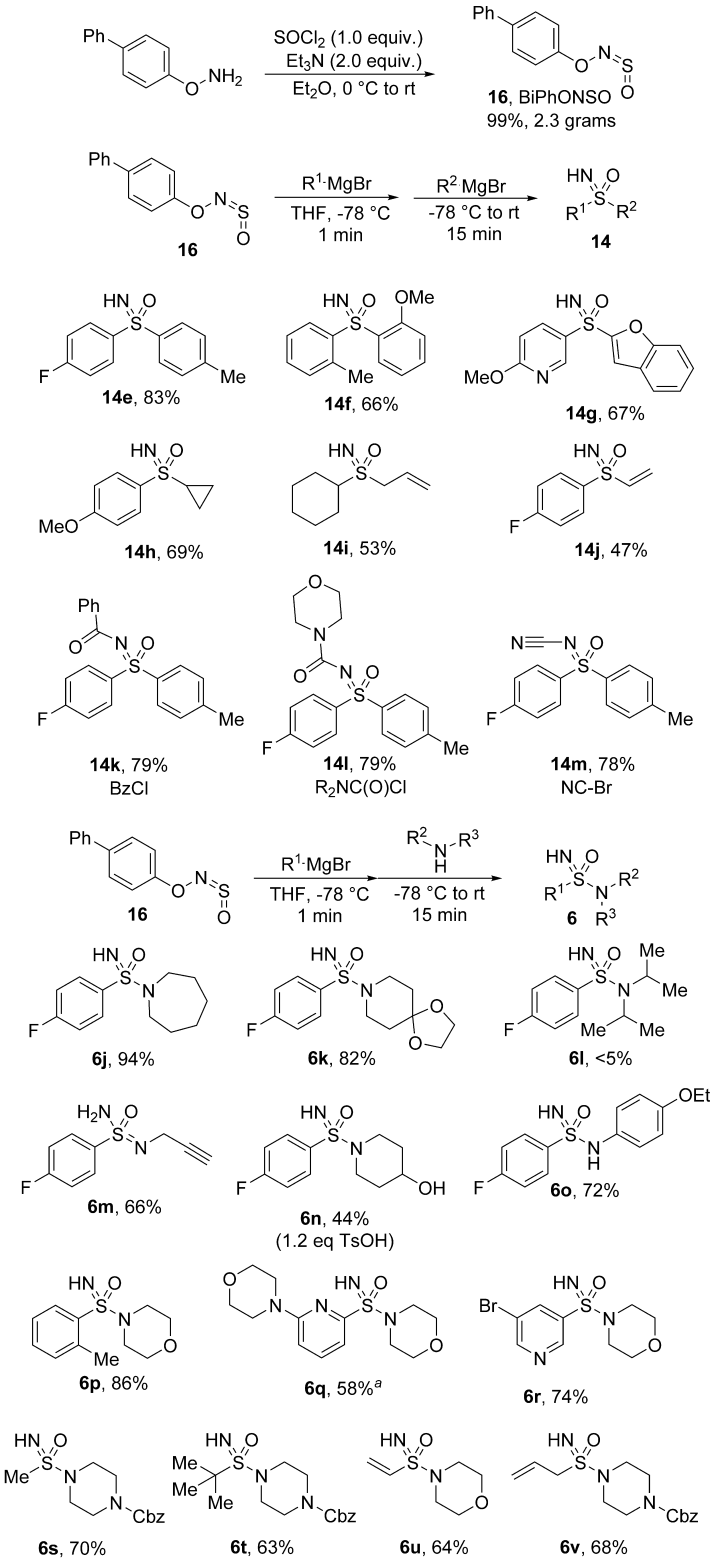
Preparation of BiPhONSO **16** and its use in the one‐pot synthesis of sulfoximines and sulfonimidamides.

Despite significant experimentation, we could not develop a direct one‐pot synthesis of sulfoximines using conventional sulfinylamine reagents such as **5** and **11**. These efforts were hindered by the poor reactivity of known sulfur(VI) electrophiles such as sulfonimidoyl chlorides, fluorides and esters with organometallic carbon nucleophiles, which often do not react or undergo reduction to the sulfinamide instead of substitution at sulfur. We hypothesised that the use of a sulfinylamine bearing a suitable leaving group on nitrogen would, after addition of a carbon nucleophile, lead to rare S‐electrophilic sulfinyl nitrene intermediates.^[30]^ It was planned that these species would react more efficiently with organolithium and Grignard reagents. We found that the *O*‐aryl‐*N*‐sulfinylhydroxylamine BiPhONSO (**16**) combines good stability and a convenient solid form with excellent reactivity.^[12]^ Addition of a Grignard or organolithium reagent to BiPhONSO initially forms an *N*‐aryloxysulfinamide, which loses the phenoxide group to give a sulfinyl nitrene. This can then react with a second carbon or nitrogen nucleophile to give sulfoximines or sulfonimidamides (Scheme 7). The scope proved broad for both classes of molecules and over 70 examples were reported in total. For sulfoximines, (hetero)aryl nucleophiles provided products in good yields (**14 g**), and steric factors did not seem to influence reactivity as evidenced by the formation of a rare doubly *ortho*‐substituted sulfoximine **14 f**. Alkyl, allyl and alkenyl organometallic nucleophiles could also be incorporated (**14 h**–**14 j**). Furthermore, since the sulfoximine product is formed as an anion, acyl, carbamoyl and cyanogen halide electrophiles could be added at the end of the reaction to give fully substituted compounds **14 k**–**14 m**.

For sulfonimidamides, secondary and primary amines and anilines were all competent as the second nucleophile. The preparation of sensitive compounds such as ketal **6 k** is notable considering that these would likely not survive the acidic conditions required to remove the trityl group in the earlier TrNSO‐based synthesis. A limitation did emerge for very bulky amines, as diisopropylamine failed, even when added as an anion (LDA). Amines containing acidic protons failed in the standard reaction, but reactivity could be recovered by addition of 4‐toluenesulfonic acid (TsOH), as was the case for secondary alcohol **6 n**. Similar to the sulfoximine synthesis, (hetero)aryl, alkyl, alkenyl and allyl organometallic reagents all worked as the first component, with excellent tolerance of steric hindrance.

**Scheme 8:**
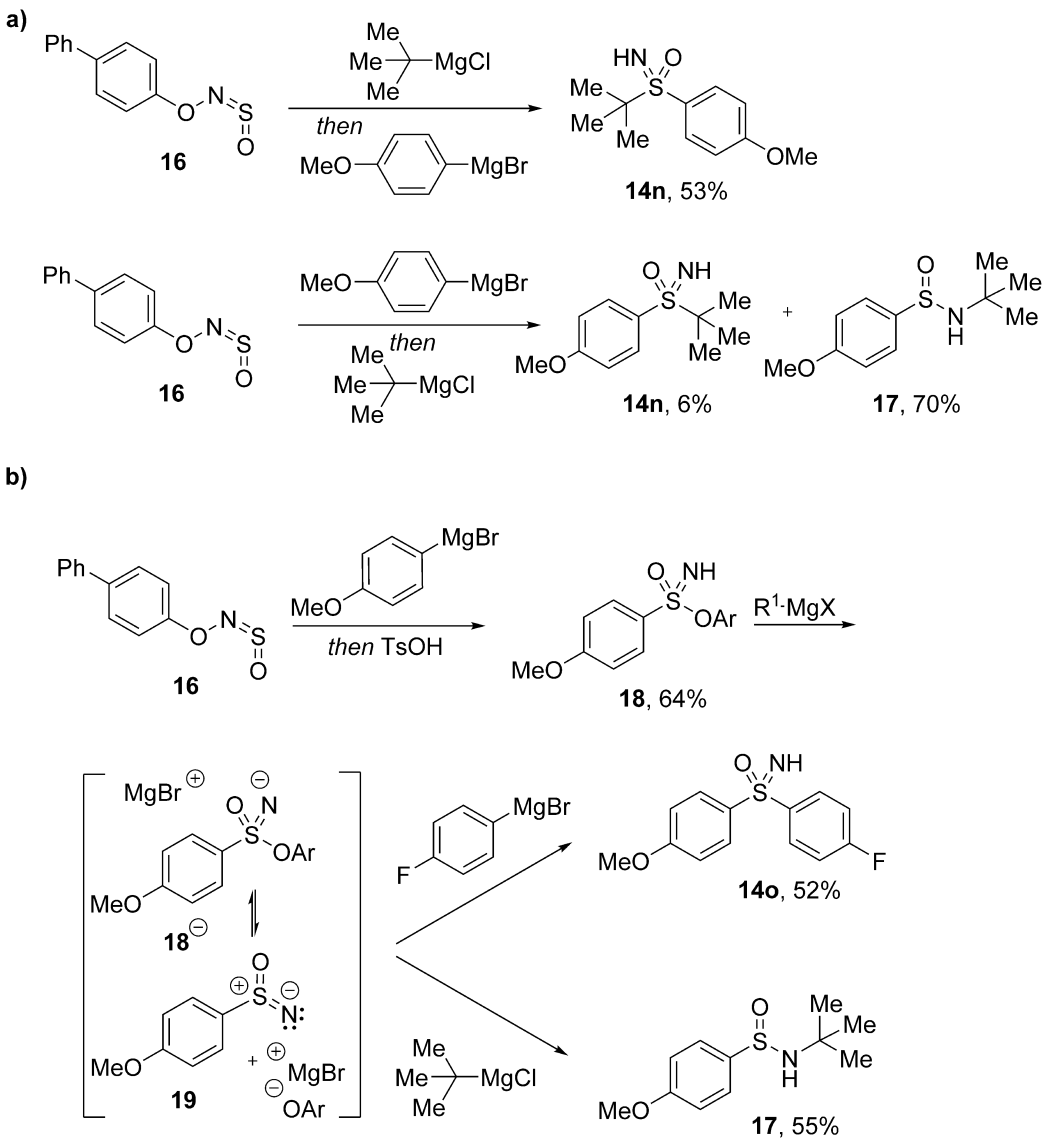
Mechanistic investigation into the synthesis of sulfoximines from BiPhONSO. a) Role of the order of Grignard reagent addition. b) Isolation of sulfonimidate ester **18** and its reaction with Grignard reagents. Ar=4‐PhC_6_H_4_.

In certain cases, the order of addition of the organometallic reagents was key. When *t*‐butylmagnesium chloride was added first with an aryl nucleophile second, the desired sulfoximine was obtained in good yield. However, when the order was reversed, the *N*‐*tert*‐butyl sulfinamide was the major product (Scheme 8). This was judged to provide evidence of the intermediacy of a sulfinyl nitrene, considering that nitrenes are well known for reactivity at nitrogen.^[31]^ For such bulky alkyl groups, it seems that addition to the central sulfur atom is disfavoured and reaction at nitrogen consequently dominates. Sulfonimidate ester intermediates could be isolated when TsOH was added after the initial carbon nucleophile, and these were found to provide the same products when 2 equivalents of the organometallic reagent were added. These results suggest that the phenoxide anion which is expelled during the reaction may add back in, and that the ester could act as a reservoir for the sensitive nitrene species. Finally, computational studies described in the paper found a plausible pathway from the starting material to the product *via* a triplet sulfinyl nitrene intermediate and agreed with experimental results that addition to sulfur was favoured over nitrogen for the second nucleophile.

**Scheme 9:**
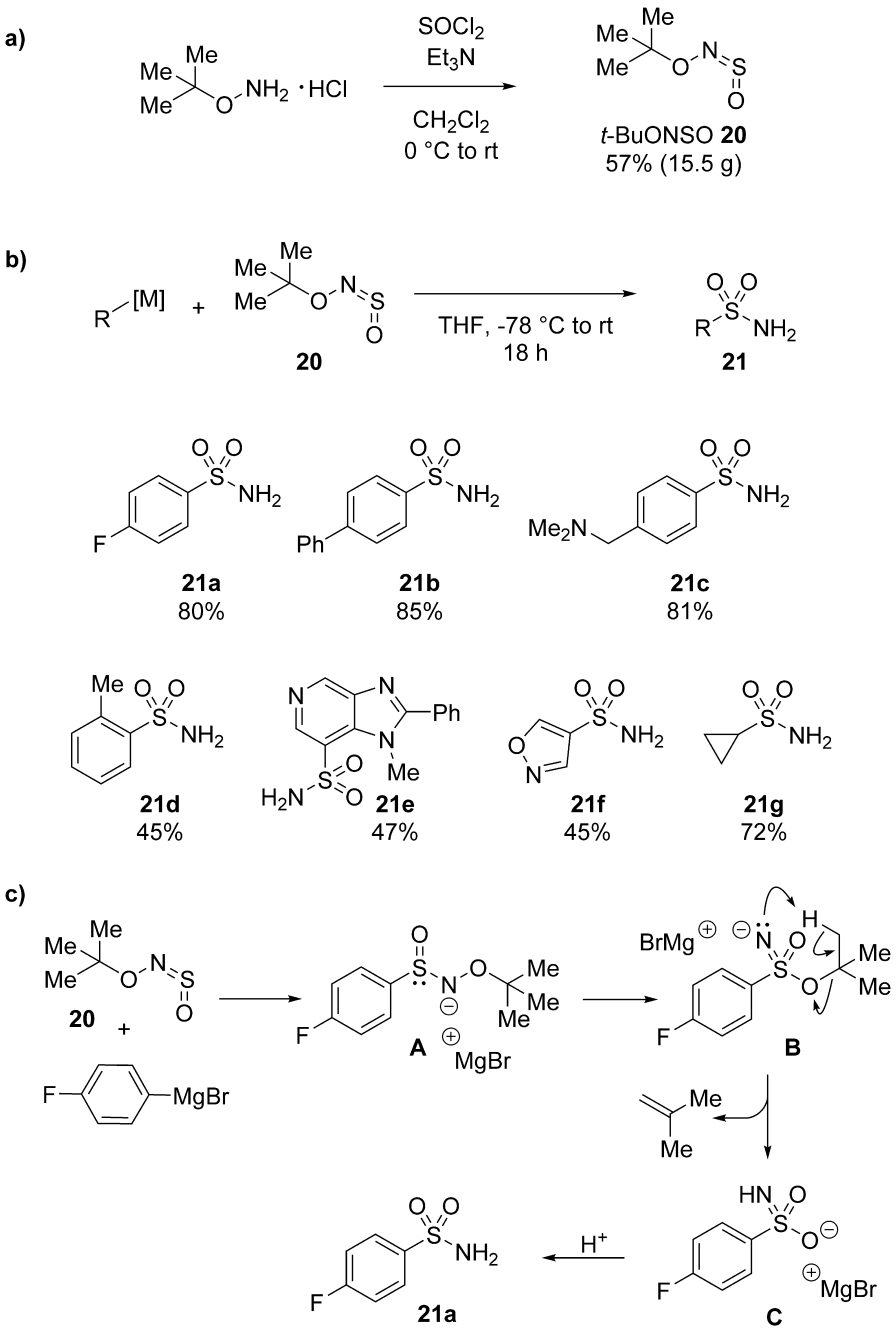
a) Decagram scale synthesis of *t*‐BuONSO **20**. b) Its reactivity with carbon nucleophiles to give primary sulfonamides **21**. c) A tentative mechanism involves rearrangement of sulfinamide anion **A** and elimination of isobutene from the resultant sulfonimidate ester intermediate **B**.

During our investigations into *N*‐sulfinylhydroxylamine reagents, we also found that the reaction of organometallic reagents with *N*‐sulfinyl‐*O*‐*tert*‐butylhydroxylamine (*t*‐BuONSO, **20**) unexpectedly gives primary sulfonamides.^[32]^ *t*‐BuONSO, a stable colourless liquid, was prepared on over 100 mmol scale by combining commercially available *tert*‐butylhydroxylamine hydrochloride with thionyl chloride and purified by distillation (Scheme 9). The reaction proved relatively insensitive to steric and electronic factors and was compatible with aryl and alkyl nucleophiles. Heterocyclic Grignard and organolithium reagents gave products in generally lower but still useful yields. The one‐pot preparation of complex, polar primary sulfonamides such as **21 e** from readily available aryl bromides, demonstrates the synthetic value of sulfinylamines to chemists in industry. Preliminary mechanistic experiments showed no incorporation of ^18^O‐labelled water in the product upon quenching, as well as identification of signals in the ^1^H NMR spectrum of a reaction aliquot consistent with isobutene. A mechanistic proposal suggested that sulfinamide intermediate **A**, formed by the attack of the Grignard reagent to *t*‐BuONSO, undergoes rearrangement to sulfonimidate ester anion **B**. Intramolecular proton transfer to eliminate isobutene followed by tautomerisation and protonation gives the primary sulfonamide products.

**Scheme 10:**
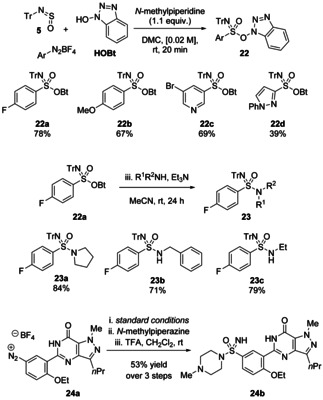
Radical reaction of TrNSO with aryldiazonium salts and HOBt to form activated sulfonimidate esters **22**, and subsequent reaction with amines to give trityl‐protected sulfonimidamides **23**.

Sulfonimidamides have also been prepared from TrNSO in a two‐step procedure by Bolm and co‐workers.^[33]^ Using a radical strategy originally developed by Wu for the synthesis of sulfonyl derivatives from SO_2_,^[34]^ *O*‐benzotriazolyl sulfonimidate esters **22** were synthesised from aryldiazonium salts, hydroxybenzotriazole (HOBt), TrNSO and *N*‐methylpiperidine in dimethylcarbonate (DMC) (Scheme 10). A good range of electron‐donating and ‐withdrawing groups were tolerated on the aromatic ring, and examples were shown for 5‐ and 6‐membered heterocycles. These activated sulfonimidate esters could then be reacted with amines at room temperature over 24 hours to give trityl‐protected sulfonimidamides (**23 a**–**23 c**). Aniline and relatively bulky amines such as *tert*‐butylamine failed to give any product. Notably, a sulfonimidamide analogue of the drug Sildenafil was prepared using the methodology (compound **24 b**). The authors suggest that the Lewis basic sulfinylamine oxygen atom plays a role in activating the diazonium salt to give an aryl radical, which then adds to the sulfinylamine.

## Use in Cycloadditions and Ene Reactions

**Scheme 11:**
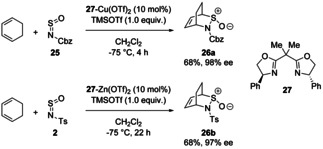
Enantioselective Diels‐Alder reaction of sulfinylamines with 1,3‐cyclohexadiene catalysed by Cu‐ or Zn‐bisoxazoline complexes.

As mentioned earlier in the review, sulfinylamines can undergo cycloadditions, principally acting as dienophiles in Diels‐Alder reactions, or dipolarophiles in [3+2] cycloadditions. This field has been covered in a previous review,^[13]^ but we highlight here a more recent asymmetric example by Gautun and co‐workers.^[35]^ Sulfinylamines substituted with Cbz (**25**) or tosyl (**2**) groups formed adducts with 1,3‐cyclohexadiene in the presence of 10 mol% of chiral bisoxazoline ligand **27** complexed to Cu(OTf)_2_ or Zn(OTf)_2_, respectively (Scheme 11). The *endo* products **26 a** and **26 b** were obtained selectively in good yields with 98 % and 97 % ee, respectively. Stoichiometric TMSOTf was required as an additive to assist catalyst turnover.

**Scheme 12:**
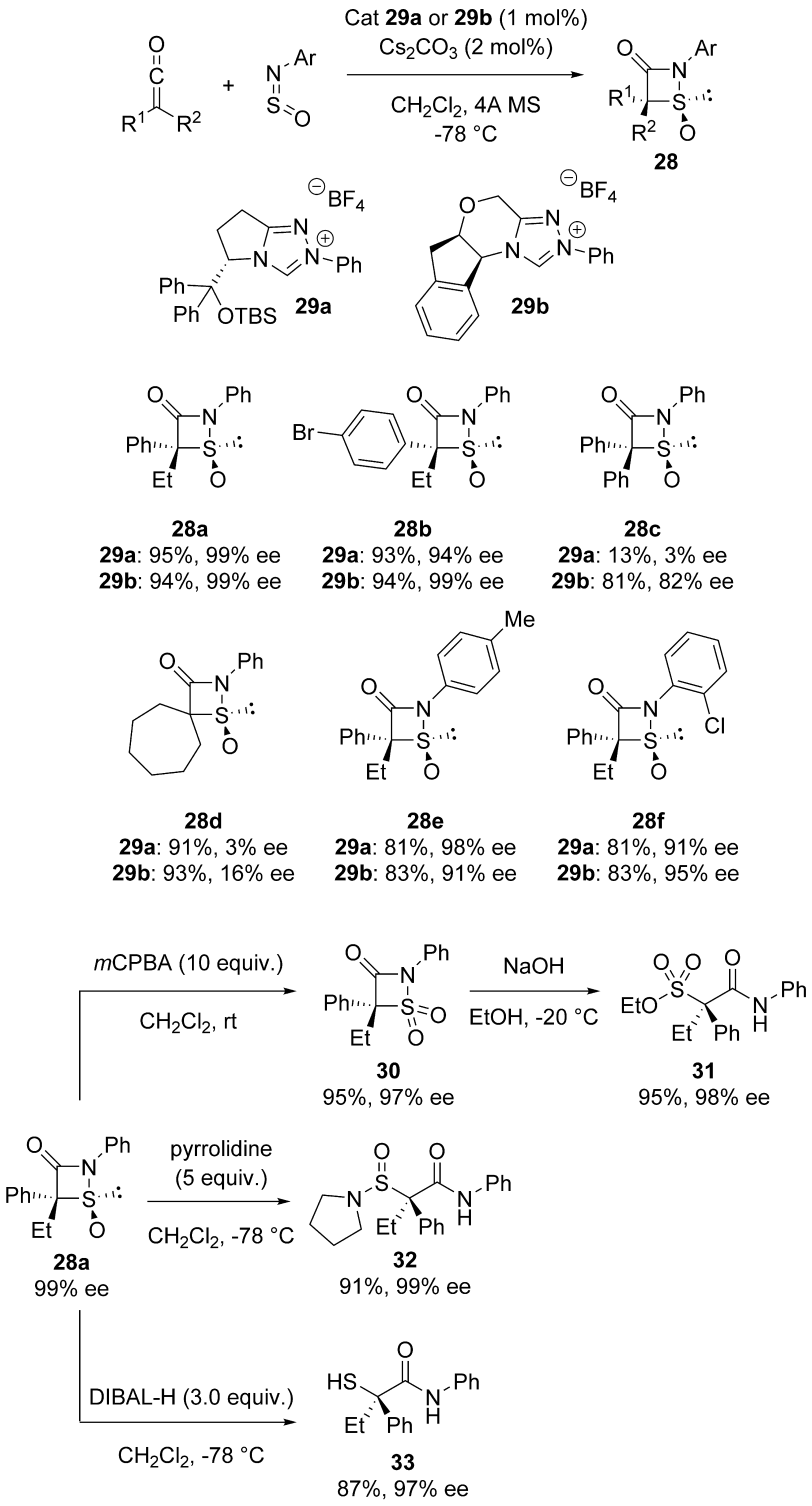
Asymmetric NHC‐catalysed cycloaddition of *N*‐sulfinylanilines and ketenes to give 4‐membered heterocycles **28** and their derivatisation to useful acyclic building blocks.

In 2011 Ye and co‐workers reported an NHC‐catalysed [2+2] cycloaddition between ketenes and *N*‐arylsulfinylamines to give enantioenriched 1,2‐thiazetidine‐3‐one‐1‐oxides **28** (Scheme 12).^[36]^ The reaction worked best with arylalkyl ketenes, giving the heterocyclic products in excellent yields and enantiomeric excess. Using a diaryl ketene, the product **28 c** was obtained in a lower 82 % ee (with catalyst **29 b**), while for a cyclic dialkyl ketene, enantioselectivity collapsed regardless of catalyst (**28 d**). However, some substitution on the *N*‐sulfinylaniline was tolerated (**28 e**/**28 f**) and the low catalyst loadings (1 mol%) are particularly noteworthy. It is also notable that the compounds could be derivatised to useful acylic products with enantioenriched quaternary centres. Product **28 a** could be oxidised to sulfone **30** with *meta*‐chloroperbenzoic acid (*m*CPBA). The S−N bond was cleaved selectively over the lactam C−N bond by the addition of ethoxide to **30** to give sulfonate ester **31**. The S−N bond could also be cleaved by the addition of pyrrolidine to **28 a** to give sulfinamide **32**, or by reduction with DIBAL‐H at −78 °C to give the thiol **33**. Mechanistically, the authors suggest that the reaction proceeds by addition of the NHC to the ketene and attack of the resulting enolate to the sulfinylamine. Cyclisation of the sulfinamide nitrogen onto the carbonyl to release the NHC then gives the exotic 4‐membered ring products.

**Scheme 13:**
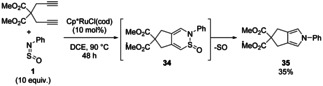
Ru‐catalysed cycloaddition of *N*‐sulfinylaniline **1** with a 1,6‐diyne.

In 2004, Yamamoto and co‐workers reported an example of a 1,6‐diyne undergoing cycloaddition with *N*‐sulfinylaniline **1** (PhNSO) in the presence of Cp*RuCl(cod) to give bicylic pyrrole **35** (Scheme 13).^[37]^ Though the yield is low and a large excess of PhNSO is required, it represents a rare example of sulfinylamines being used in metal catalysis and shows their potential for use in aromatic heterocycle synthesis.

**Scheme 14:**
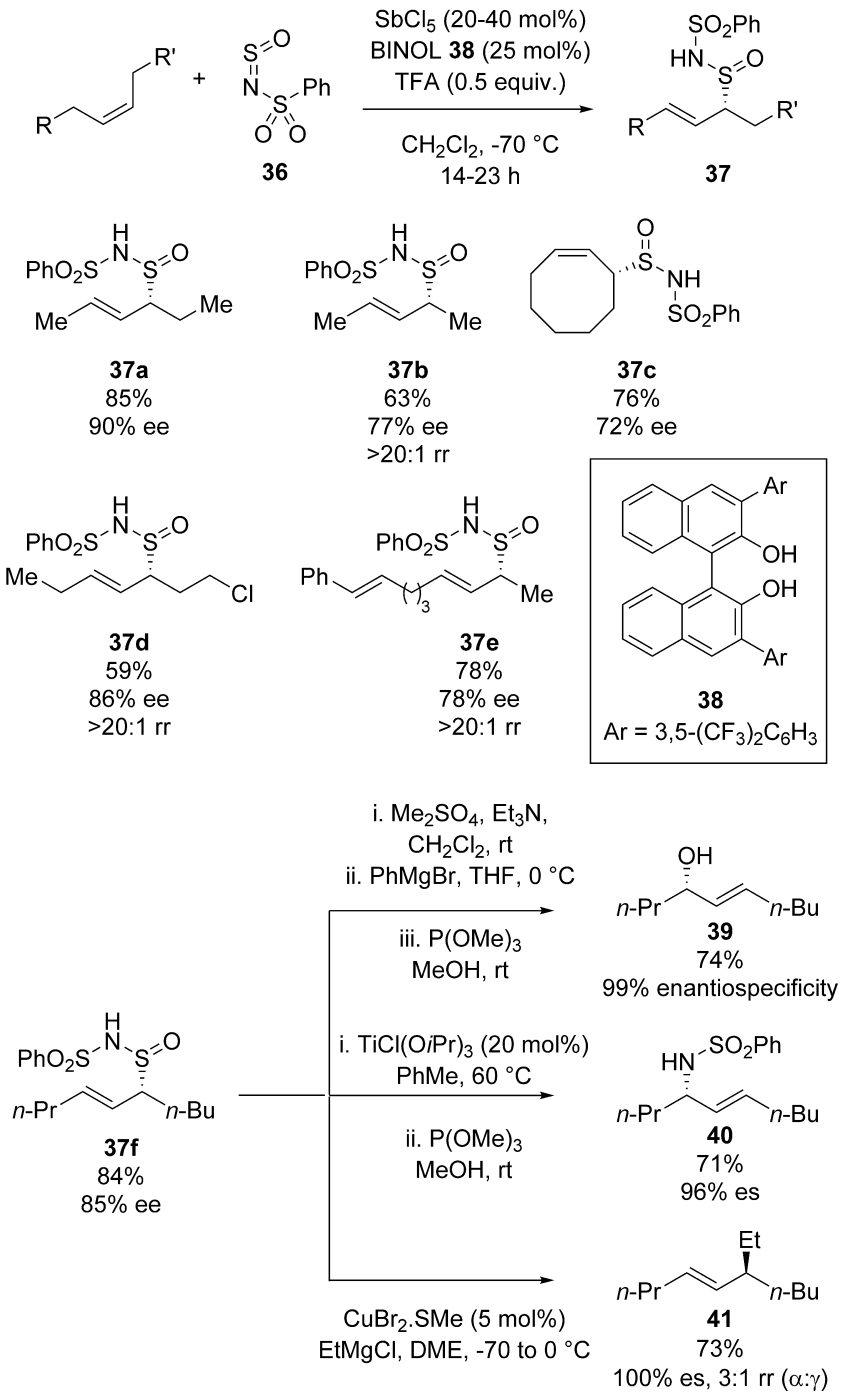
Enantioselective allylic oxidation of unactivated (*Z*)‐alkenes using *N*‐sulfinylsulfonamide **36** and derivatisation of the resulting allyl sulfinamides to diversely substituted chiral products.

In 2017, Tambar and co‐workers reported a method for asymmetric allylic oxidation *via* the hetero‐ene reaction of *N*‐sulfinylbenzenesulfonamide **36** with unactivated internal (*Z*)‐alkenes to give allylsulfinamide products **37** (Scheme 14).^[38]^ Good levels of enantioselectivity were attained by conducting the reaction at −70 °C in the presence of antimony pentachloride and chiral BINOL co‐catalyst **38**. Crucially, no reaction occurred at this temperature without the catalyst. The products could be derivatised enantiospecifically to form C−O, C−N and C−C bonds (**39**–**41**), demonstrating the utility of the enantioenriched sulfinamide products. Reduction of **37 f** to the thiol and conversion to an allylic chloride were also shown in the paper. The authors propose that the Brønsted acid catalyst formed by BINOL **38** and SbCl_5_ activates the sulfinylamine *via* a hydrogen bonding interaction to the sulfinyl oxygen, lowering its lowest unoccupied molecular orbital and facilitating the ene reaction. Overall, the paper represents an important expansion of the chemistry of sulfinylamines, showing that they may be used to “upgrade” hydrocarbon feedstocks to higher value chiral products *via* enantioselective catalysis, accessing useful compounds that do not even necessarily contain a sulfur atom.

## Conclusions

This Minireview has described the properties of sulfinylamines and their applications in organic synthesis over the last decade. They have enabled significant advances in the synthesis of medicinally important sulfur compounds such as sulfoximines and sulfonimidamides, as well as undergoing enantioselective cycloadditions to give functionalised heterocycles and asymmetric allylic oxidations of simple alkenes. The development of stable (moisture‐insensitive) reagents such as TrNSO **5** has been an important step in encouraging wider use of sulfinylamines by pharmaceutical companies,^[27]^ and other academic groups.^[33]^ The discovery that *N*‐sulfinylhydroxylamines (BiPhONSO **16**) can function as precursors to highly reactive and electrophilic sulfinyl nitrenes, which has allowed the first one‐pot *de novo* synthesis of sulfoximines,^[12]^ will likely also have an impact on the way organosulfur molecules are synthesised in future. However, the scarcity of sulfinylamines in the organic chemistry literature is also worthy of comment. As a result of the dormancy of sulfinylamines over the last several decades, few or no methods have been developed which exploit them in transition metal, organo‐, photoredox or electrocatalysis. It is, therefore, likely that new and unexpected reactivity modes of these compounds are waiting to be discovered. The virtual absence of methods to transform sulfinylamines, which are prochiral, directly to enantioenriched sulfinamides or chiral sulfur(VI) compounds with control of the emergent sulfur stereocentre is also notable. For preparative‐scale chemistry, enantioenriched sulfinamides are generally made starting from chiral menthyl sulfinate esters (*via* resolution of diastereomers),^[39]^ or more recently from stereospecific de‐alkylation of *S*‐*tert*‐butylsulfoximines (often made from commercially available *tert*‐butanesulfinamide).[^40^, ^41^, ^42^] Other ways to access enantioenriched sulfur(VI) compounds are also appearing in the literature, including other chiral auxiliary approaches,^[43]^ kinetic resolution[^44^, ^45^] and enantioselective desymmetrisation^[46]^ of sulfoximines. However, the possibility of more direct approaches combining sulfinylamines with the many chiral Lewis acids and bases, organocatalysts, ligands and metal catalysts that have been developed in recent decades will surely not evade the attention of synthetic chemists for much longer. We hope that by showcasing their recent applications and the discovery of stable reagents, this Minireview will act as a clarion call for the development of new chemistries which capitalise on the special reactivity and properties of sulfinylamines.

## Note added in proof:

**Scheme 15:**
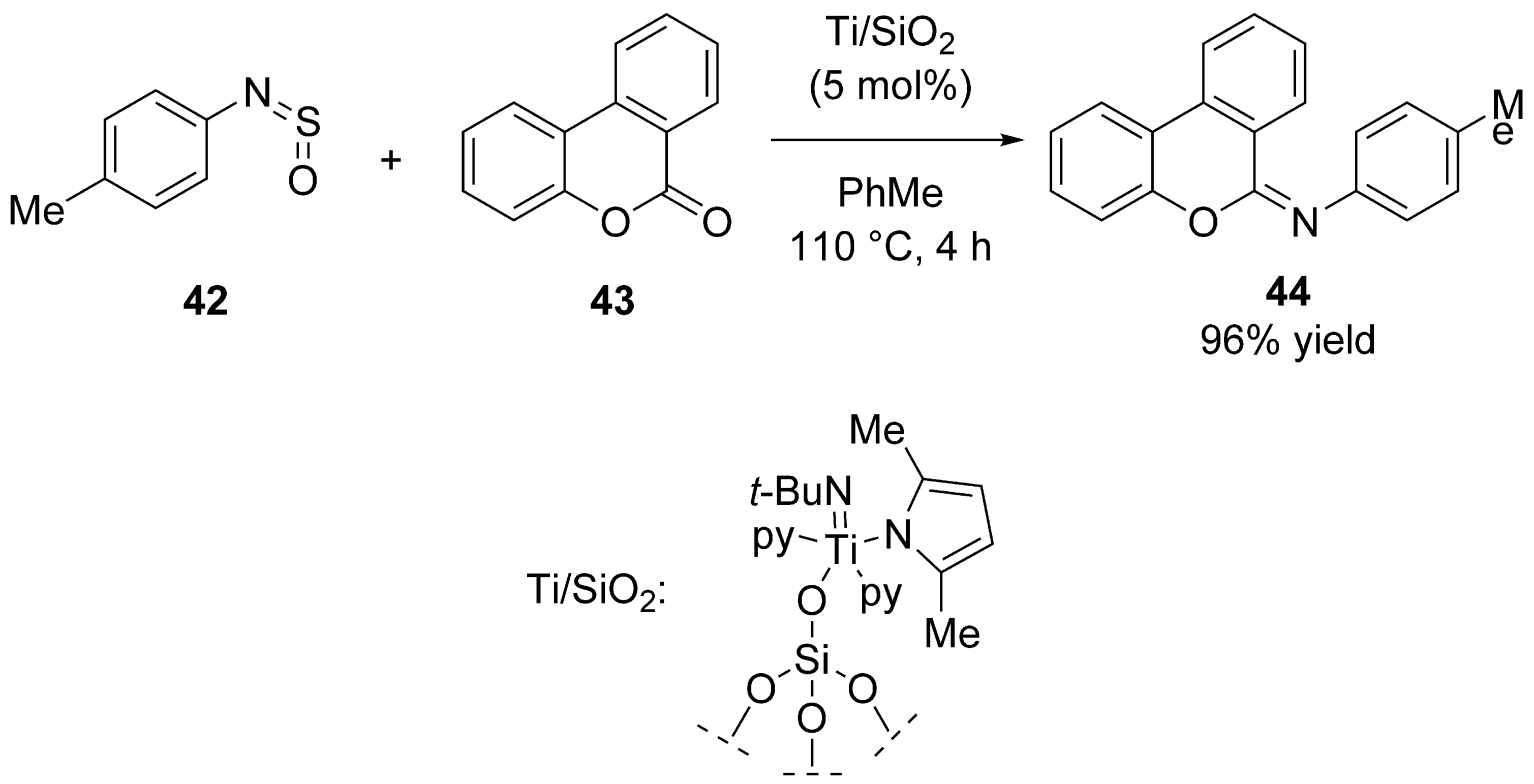
Direct imidation of lactones to imidates using SiO_2_‐supported titanium imido complexes.

Following submission of our manuscript, the first direct imidation of lactones **43** to cyclic imidates **44** was reported using *N*‐arylsulfinylamines and a silica‐supported titanium imido complex (Ti/SiO_2_).^[47]^ A representative example is shown (Scheme 15). This builds on earlier work using sulfinylamines as imido‐transfer reagents.[^48^, ^49^, ^50^]

## Conflict of interest

The authors declare no conflict of interest.

## Biographical Information

*Thomas Davies obtained an MChem degree from the University of Glasgow in 2014, spending one year on an industrial placement at F. Hoffmann‐La Roche (Basel). He completed his PhD (2018) and a 9 month postdoctoral stay with Prof. M. C. Willis at the University of Oxford working on organosulfur chemistry. He is currently an Alexander von Humboldt postdoctoral fellow in the group of Prof. A. Fürstner at the Max‐Planck‐Institut für Kohlenforschung*.

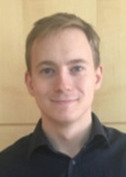

## Biographical Information

*Michael Willis received his undergraduate education at Imperial College London and his PhD from the University of Cambridge working with Prof. S. V. Ley, FRS. After a postdoctoral stay with Prof. D. A. Evans at Harvard University, he was appointed to a lectureship at the University of Bath in November 1997. In January 2007 he moved to the University of Oxford, where he is now a Professor of Chemistry and a Fellow of Lincoln College. His group‘s research interests are based on the development and application of new catalytic processes for organic synthesis*.